# Herbal Medicine: Enhancing the Anticancer Potential of Natural Products in Hepatocellular Carcinoma Therapy Through Advanced Drug Delivery Systems

**DOI:** 10.3390/pharmaceutics17050673

**Published:** 2025-05-20

**Authors:** Ghazala Muteeb, Manar T. El-Morsy, Mustafa Ali Abo-Taleb, Salma K. Mohamed, Doaa S. R. Khafaga

**Affiliations:** 1 Department of Nursing, College of Applied Medical Sciences, King Faisal University, Al-Ahsa 31982, Saudi Arabia; 2 Bio-Nanotechnology Department, Faculty of Nanotechnology, Cairo University, Giza 12613, Egypt; 14222023442600@pg.cu.edu.eg; 3 Biotechnology Department, Faculty of Science, Cairo University, Giza 12613, Egypt; 201928373@std.sci.cu.edu.eg; 4 Faculty of Medicine, Galala University, Suez 43511, Egypt; salma.khaled@gu.edu.eg; 5 Health Sector, Faculty of Science, Galala University, Suez 43511, Egypt

**Keywords:** nanomedicine, HCC, liver cancer, natural products, drug delivery, targeted therapy

## Abstract

Hepatocellular carcinoma (HCC) is an aggressive and prevalent liver cancer with a poor prognosis. Nanotechnology combined with natural products has emerged as a promising strategy to enhance HCC treatment efficacy. This review assesses the current literature on the application of nanotechnology in delivering natural products for HCC therapy. A comprehensive search was conducted in PubMed, Science Direct, Web of Science, and Google Scholar to identify relevant studies published up to the present articles focusing on nanotechnology-based drug delivery systems using natural products for HCC therapy, including different nanoparticle (NP) formulations and therapeutic interventions, were included. Natural products with anticancer properties have been encapsulated using various nanocarriers such as liposomes, polymeric nanoparticles, and quantum dots, which have improved drug stability, prolonged circulation time, and enhanced targeted delivery to HCC cells. These advancements have led to increased therapeutic efficacy and reduced side effects. Additionally, combining multiple natural products or integrating them with conventional therapies via nanocarriers enables personalized treatment approaches based on patient characteristics and molecular profiles. The integration of nanotechnology with natural products shows great potential for improving HCC treatment outcomes, representing a significant advancement in precision medicine for liver cancer and paving the way for more effective and personalized therapeutic strategies.

## 1. Introduction

Global epidemiological trends in liver cancer incidence exhibit significant geographical variation [1]. Recent surveillance data indicate a notable increase in incidence rates in economically developed countries, especially in Western Europe, North America, and Oceania [1]. In contrast, various traditionally high-risk Asian countries have indicated decreasing rates, implying differing effects of preventive strategies, environmental factors, and healthcare interventions among regions [1]. The male-to-female rate ratios also vary across regions, with higher ratios observed in France and Egypt compared to sub-Saharan African and South American registries [2]. The global burden of liver cancer is anticipated to rise significantly, with a notable increase in new cases and deaths expected by 2040, especially in Asia [3]. As the third most common cause of cancer-related death worldwide, liver cancer ranks as the sixth most common type of cancer. In 2020, there were expected to be 830,180 fatalities and 905,677 new cases globally [4,5], and the rate of liver cancer escalated with advancing age [5]. It is estimated that HCC accounts for 80% of occurrences of liver cancer [4,5]. Most HCC cases have chronic liver disease, and most of these cases have cirrhosis as a contributing factor [6]. Alpha-fetoprotein (AFP) levels and ultrasonography are the primary methods used in HCC screening. Magnetic resonance imaging (MRI) or computed tomography (CT) is used to diagnose abnormalities. Nevertheless, pathological diagnosis is necessary in a small number of cases [7].

Furthermore, the most common cancer treatment approaches used worldwide for HCC therapy are traditional therapeutic techniques, which include radiation, chemotherapy, and surgical procedures [8,9,10]. These approaches have certain limitations, including non-specific biological distribution and non-targeted therapeutic delivery with an inadequate dosage of anticancer agents in the selected tumor microenvironment and some biologic imaging and diagnosis constraints [11]. Also, traditional chemotherapy and natural products have different therapeutic efficacy in cancer treatment due to differences in toxicity, resistance, and mechanisms of action [12,13]. Chemotherapy drugs such as cisplatin and doxorubicin are beneficial, but they frequently lead to drug resistance and severe damage in many organs [13,14]. Resistance to chemotherapy is a major concern, resulting in 80–90% of deaths caused by cancer [15]. Natural products can improve chemotherapy efficiency by sensitizing cancer cells and overcoming chemoresistance [16,17].

On the other hand, recent studies have reported a variety of different natural products for the possible treatment of HCC such as resveratrol, curcumin, and ginger extract, have been investigated as safe and effective anticancer agents with potentially fewer side effects [18,19,20,21]. Also, natural products have shown underlying molecular mechanisms involving apoptosis, anti-metastasis, and anti-angiogenesis [22,23,24]. Nanotechnology plays a significant role in the treatment of HCC. It offers targeted and precise therapy, overcoming the limitations of conventional cancer treatments such as chemotherapy, radiation, and surgery. Nanoparticles can be engineered to specifically target cancer cells, delivering drugs directly to them and minimizing damage to healthy cells [25,26,27]. Functionalized nanomaterials have been developed to embolize tumor vasculature, achieve high drug loading efficacy, enable tumor targeting, and provide controlled sustained release of drugs [28]. These nanoparticles can control drug release and enhance drug stability; furthermore, they improve drug accumulation in the target tumor because they can specifically enter neoplastic sites through passive and/or active pathways, therefore reducing drug toxicity [29,30]. Natural products with nanocarriers have shown promise in HCC therapy [31]. These nanocarriers can encapsulate bioactives from medicinal plants, allowing for targeted delivery to tumor areas with minimal damage to healthy cells [32]. Furthermore, using natural bioactive-loaded nanocarriers has shown promising therapeutic efficacy in HCC treatment, providing targeted delivery of natural active ingredients. Overall, natural products offer a potential alternative to traditional chemotherapy in the treatment of HCC, with the advantage of potentially lower toxicity and multi-level effects. This review aims to focus on the effective role of delivery systems to enhance the anticancer effect of natural products to treat HCC without side effects on normal cells.

## 2. Methodology

A comprehensive search was conducted in PubMed, Science Direct, Web of Science, and Google Scholar to identify relevant studies published on the role of nanotechnology-based drug delivery systems using natural products for HCC therapy. The search included studies published from 1976 to 2025 using the following keywords: ‘nanotechnology’, ‘drug delivery systems’, ‘natural products’, ‘hepatocellular carcinoma’, and ‘cancer therapy’. Inclusion criteria encompassed peer-reviewed articles and preclinical trials that investigated the therapeutic potential of natural product-based nanocarriers in HCC treatment. Studies were excluded if they lacked a focus on nanotechnology or did not provide sufficient data on the efficacy of natural products in HCC therapy.

## 3. Liver and Its Disorders

Necrosis of the liver cells and their incapacity to regenerate are signs of liver damage. This poses a serious risk to patients’ lives and health because, in extreme cases, it can cause bleeding and liver failure [33]. Additionally, astaxanthin has been demonstrated to lessen liver damage brought on by a number of environmental pollutants, such as acetaminophen, carbon tetrachloride, and lipopolysaccharide [34]. High-fat, restricted-protein diets have been associated with liver damage as well as notable alterations in the gut microbiota, which include a decrease in Lactobacillus abundance and an increase in the Firmicutes/Bacteroidetes ratio. A larger bile acid pool and compositional changes, particularly higher levels of CDCA and LCA species, were also linked to these dietary modifications. The liver’s adaptive regulation of bile acid synthesis and transport, as well as the FXR-SHP pathway, was necessary to preserve the homeostasis of bile acid and hepatic lipid metabolism. By activating the FXR pathway, CDCA was found to lessen endoplasmic reticulum stress brought on by palmitic acid in HepG2 cells [35]. Last but not least, it is crucial to remember that a number of established risk factors, such as age, cirrhosis, sex, and co-infection with HIV or other hepatitis viruses like hepatitis C and D, are linked to HCC in people with chronic hepatitis B [36].

## 4. Hepatoprotective Natural Products

Natural products obtained from medicinal plants, including phenolics, flavonoids, alkaloids, and polysaccharides, are crucial in the treatment of HCC in response to their various bioactive properties [37,38,39]. Hence, natural products exhibit significant therapeutic potential by targeting many molecular pathways associated with the development of cancer [38,40]. Phenolic and flavonoid compounds exhibit anticancer activity via regulating signaling pathways, including STAT3, NF-κB, and CXCR4, while also influencing the tumor environment to improve the immune system [41,42]. Alkaloid substances, derived from traditional Chinese medicine, demonstrate anti-hepatocarcinogenic properties by suppressing proliferation, metastasis, and angiogenesis while enhancing the autophagy and apoptosis processes [40]. Furthermore, natural polysaccharides indicate the ability to minimize the adverse effects of chemotherapeutic agents and possess anti-HCC properties through mechanisms such as apoptosis and immune system stimulation [43]. Thus, phenolics, flavonoids, alkaloids, and polysaccharides from medicinal plants modulate important signaling pathways and enhance the immune response in treating HCC, making them valuable in developing new HCC therapies. The following section describes different natural products that are utilized in treating HCC, which have some advantages and disadvantages, as shown in Table 1.

### 4.1. Shikonin

Shikonin is a natural product that possesses anticancer properties and has been investigated for its potential use in HCC treatment [44]. Recent studies have demonstrated that shikonin has been shown to control pyruvate kinase type M2 (PKM2), which in turn causes apoptosis and inhibits proliferation and glycolysis in HCC cells [44,45,46]. Shikonin exhibits a dose-dependent effect in enhancing cell apoptosis and autophagy by reducing the expression of pyrroline-5-carboxylate reductase 1 (PYCR1) and inhibiting the PI3K/AKT/mTOR signaling pathway [22,46]. Overall, shikonin has considerable anticancer effects in HCC via various mechanisms, including PKM2 suppression, ROS-mediated apoptosis induction, and regulation of essential signaling pathways [45,47]. Lin et al. demonstrated that shikonin, with an IC_50_ value of 15 μM, causes cytotoxicity in HER2-enriched cells by stopping the cell cycle in the sub-G1 phase and triggering apoptosis within a day of treatment. Shikonin controls cell proliferation and death by regulating 38 differentially expressed genes [48]. These findings emphasize shikonin’s potential as a significant enhancement of current HCC treatments.

### 4.2. Fucoidan

Fucoidan, a naturally sulfated polysaccharide found in brown seaweed, has been investigated for its potential role in the treatment of HCC [49]. Studies have demonstrated that fucoidan inhibits the progression of HCC by a variety of pathways, including up-regulation of LINC00261, suppression of invasion, induction of apoptosis, and enhancement of cancer immunity [50]. Also, fucoidan has been shown to exhibit anticancer properties in both in vivo and in vitro investigations, showing its potential as a therapeutic drug for HCC [51].

### 4.3. Nigella sativa

*Nigella sativa*, also known as black seed, has been investigated for its potential properties against HCC [52,53]. Recent studies showed that *Nigella sativa* seed oil inhibits cellular proliferation and induces apoptosis in HCC cells by various mechanisms, including the p53 pathway and reactive oxygen species (ROS)-dependent mitochondrial apoptosis. *Nigella sativa* is considered a chemopreventive therapy due to its antioxidant mechanism in HCC and inhibition of HCC signaling pathways [54]. Moreover, thymoquinone is a principal bioactive compound of *Nigella sativa*, which has demonstrated the ability to induce apoptosis in cancer cells via multiple pathways. Furthermore, thymoquinone (TQ) has been identified as a modulator of essential signaling pathways, including the AKT pathway, which plays a vital role in the regulation of cell survival and death [55,56]. Studies indicate that *Nigella sativa* may inhibit metastasis in multiple cancer models [55]. In vivo, research indicated that the use of *Nigella sativa* essential oil significantly lowered tumor volume and avoided the development of metastasis in mice models. This indicates a possible function in inhibiting the migration of HCC to other organs [55,57]. Although *Nigella sativa* is usually thought to be safe, it can produce gastrointestinal problems and allergic reactions in particular individuals. High doses may cause toxicity, especially if combined with different drugs [58,59]. Despite its possible advantages, the use of *Nigella sativa* in the treatment of HCC is limited due to a lack of clinical data, potential toxicity, complex mechanisms of action, and obstacles to regulation. Additional investigation is needed to address these limitations and develop clear guidelines for clinical use in HCC therapy.

### 4.4. Curcumin

Curcumin has demonstrated significant outcomes, such as preventing the growth of HCC by several mechanisms. Studies have shown that curcumin inhibits the expression of vascular endothelial growth factor (VEGF), the PI3K/AKT signaling pathway, and microRNAs such as miR-21, resulting in reduced cell proliferation and elevated cell death in both in vitro and in vivo HCC models [20]. It has been demonstrated that curcumin inhibits the growth of HCC by blocking the Wnt/β-Catenin signaling pathway, causing cell cycle arrest, resulting in death [60]. Furthermore, curcumin exhibits crucial properties due to its anticancer, anti-inflammatory, and antioxidant properties [61]. Despite the potential characteristics of curcumin in HCC therapy, there are challenges to applying curcumin in clinical application, such as instability, poor bioavailability, and hydrophobicity [62]. Before curcumin is used therapeutically, these drawbacks can be resolved by functionalizing it using nanoparticles and phospholipid complexes [20,62]. Addressing these difficulties through novel studies and research is critical for achieving the full therapeutic potential of curcumin in HCC therapy.

### 4.5. Green Tea Catechins

Green Tea Catechins (GTCs), specifically Epigallocatechin-3-gallate (EGCG), have shown promise in both the prevention and the therapy of HCC, which is considered an aggressive type of liver cancer [63]. EGCG has been shown to inhibit the growth and spread of HCC by inducing apoptosis, regulating autophagy, and functioning as an anti-angiogenic agent [63,64]. EGCG inhibits human HCC cell growth by inhibiting IGF-1R phosphorylation, triggering Caspase-9 and Caspase-3, suppressing Bcl-2 and COX-2, and regulating signaling processes like NF-κB and ERK1-2 [64]. The beneficial impacts of GTC intake have been investigated in a variety of animal models, with results supporting their potential to inhibit HCC development, proliferation, and apoptosis. GTCs may decrease hepatocyte stem cell development and affect epigenetic expression in the hepatocytes [65]. Overall, recent studies suggest that GTCs, particularly EGCG, have the potential to prevent and treat HCC by targeting several pathways associated with tumor development and progression.

**Table 1:** Advantages and disadvantages of some natural plants used for HCC treatment.

Natural Product	Advantages	Disadvantages	Reference
Shikonin	Suppresses growth, invasion, migration, and metastasis. Viability of tumor cells by interacting with important signaling pathways such as PI3K/AKT, MAPK, and PKM2.	Low aqueous solubility. Hazardous (particularly when injected intravenously or intra-peritonially).	[47,66,67]
Fucoidan	It may trigger HCC cells to: Induce apoptosis. Arrest cell cycles.	Molecular structure variation. Fluctuating composition of several fucoidan preparations.	[68,69]
*Nigella sativa*	Promotes HCC cell apoptosis. Arresting the cell cycle. Suppresses the following in a dose-dependent manner: invasion, motility, and proliferation in HCC cells.	Allergic reactions. Gastrointestinal disturbances.	[70,71,72]
Curcumin	Blocking critical signaling pathways in HCC, such as STAT3/VEGF/HIF-1α. Prevent tumor growth and angiogenesis. Cell cycle arrest, especially in the S phase.	Low bioavailability.	[62,73]
Green Tea Catechins	Antioxidant properties. Exhibit hepatoprotective characteristics. Have low toxicity compared to other chemotherapies.	May cause adverse consequences. Potential risk of liver damage.	[74]

## 5. Challenges of Using Nature Products as Therapeutic Agents for HCC

The toxic effects of plants used in HCC therapy are a concern. However, medicinal plants include bioactive compounds that can target neoplastic pathways, and nanoparticle-based drug delivery systems can improve selectivity while minimizing damage to healthy cells [75,76]. Many natural products have failed to advance to the clinical trial stage due to insufficient information available about their pharmacokinetics and toxicity towards normal cells [77]. Several natural products, such as curcumin and resveratrol, have poor solubility, fast metabolism, and instability under physiological conditions, thereby limiting their therapeutic effectiveness [75,78]. For instance, the limited bioavailability of curcumin constrains its therapeutic application, despite its promising preclinical efficacy against HCC [78,79]. Furthermore, various plant-derived biologically active substances have low aqueous solubility, cellular absorption and bioavailability, causing clinical translation challenges [80,81]. Despite these limitations, natural products have the potential to be used as an alternative or complementary therapy in conjunction with current HCC chemotherapies [22,76]. Additional study is required to address the limitations and assess the safety and efficacy of plant-based treatments for HCC therapy [22,82,83].

## 6. Role of Nanotechnology in HCC Treatment

Systemic medication, transplantation of the liver, transarterial chemoembolization, surgical procedures, and excision are among the available forms of treatment [84,85]. Since most patients with liver cirrhosis have infrequent screenings and HCC is frequently asymptomatic in its early stages, the majority of HCC patients are detected in the intermediate or advanced stages [84]. Due to variables including late detection, quick development, poor response to treatment at the advanced stage, and easy recurrence, the five-year survival rate for HCC is just 20% [86]. Therefore, the necessity for an enhanced or novel diagnostic and therapeutic approach is critical. Research in biological nanotechnology has steadily increased recently [87,88,89]. Its use in the diagnosis and treatment of HCC has also been taken into account [90,91,92]. The best treatment for liver cancer is surgical excision, although only 15% of patients respond to this approach [93,94]. Chemotherapy is yet another important therapeutic approach for HCC patients. Chemotherapy is a general treatment that can impact cancerous and normally functioning cells alike. The development of multidrug resistance (MDR) and the uneven biodistribution of chemotherapeutic drugs at the tumor site are two further drawbacks [95]. Existing therapeutic techniques for HCC are not desired, highlighting an urgent need for the development of innovative approaches such as therapeutic antibodies. This is because most HCC patients have been diagnosed in more advanced stages in which existing standard chemotherapies are insufficient [96]. Accordingly, targeted therapy has been proposed for highly expressed proteins in cancer cells that have low expression in normal liver tissue. By using nanostructures to achieve specific therapeutic activities, nanotechnologies have had a major impact on the medical industry in the past few years [87,88,97,98]. Nanoparticles’ high specific surface area gives them various benefits over other molecular conjugates in terms of focused diagnostics and therapies: (1) they can have several targeting ligands, which are capable of binding to receptors on the surface of cells more affinitive; (2) they are able to carry a lot of molecules with effects and guard them from degradation; for instance, lipiodol-bridged Indocyanine Green (ICG) nano emulsion is capable of protecting the fluorochrome ICG from deterioration for a few days via its shielding effect, while the fluorescence levels of free ICG solution quickly drops to zero under identical conditions [99]; (3) they are adequate to hold different kinds of effector molecules; as well as (4) effector molecules can be released from nanoparticles in a way that best suits their mode of action [99]. Because of these benefits, nanomaterials can minimize the negative effects of anticancer medications while simultaneously increasing the sensitivity of biomarker or image detection and facilitating targeted drug delivery [99,100]. Researchers working on nanomedicine are attempting to improve medical constraints by the application of nanotechnology. A therapeutic agent or screening factor and a system for delivering it comprising a targeting moiety, nanocarrier, and stimuli-responsive units are the two parts needed to build a novel therapeutic or image device [101,102,103,104]. The development of a cleverly targeted nanocarrier necessitates a thorough understanding of the physicochemical properties of nano-based materials, as well as the properties of the targeted tissue and how they behave biologically when entering the body. Taking all of these into account, a therapeutic instrument based on nanotechnology may be developed, allowing for the carefully regulated delivery of cargo to target cells in response to certain stimuli while having no negative effects on healthy cells [105,106,107]. Nanotechnology has created two main goals in the realms of diagnostics and therapy for overcoming these drawbacks: effective delivery to reach adequate levels in targeted tumor areas without negative effects or little damage to normal cells, and selective and specific targeting [55]. In order to avoid the liver’s fast evacuation and to precisely address the HCC microenvironment, greater emphasis needs to be paid to the architectonics of nanostructures for either active or passive transport to the liver [108]. When it comes to passive targeting, the liver acquires more NPs than other organs after intravenous administration. The modulation of blood circulation lifespan is influenced significantly by surface modification of nanocarriers, leading to alterations in their physical, chemical, and biological identities [108,109]. To ensure proper biological distribution and effective delivery, a targeting moiety such as immunotoxins, radioimmunotherapeutics, and biocompatible and biodegradable carriers is critically needed [55,109]. For instance, tyrosine kinase inhibitors (TKIs) used to treat advanced HCC, such as staurosporine and sorafenib (SFN), have side effects similar to those of other chemotherapy drugs that affect normal cells and increase resistance to TKIs [110,111]. Thus, in order to overcome cancer cells’ resistance to anticancer TKIs, focused novel TKIs need to be developed with the ability to potentially target and inhibit particular signaling pathways in cancer cells [111]. Since some TKIs are quite harmful when used alone, nanomedicine handles them to increase TKI levels in tumors in a targeted, safe manner [110,111]. The use of several nanomedicines for HCC treatment is discussed in this review. Next, the readers are introduced to the nanocarriers used in HCC diagnosis and treatment, such as lipid, polymeric, and inorganic nanostructures. Lastly, emphasis is placed on the clinical translation and biological safety of nanomedicines in the identification and management of HCC.

## 7. Green Synthesis of Nanoparticles

**Figure 1:**
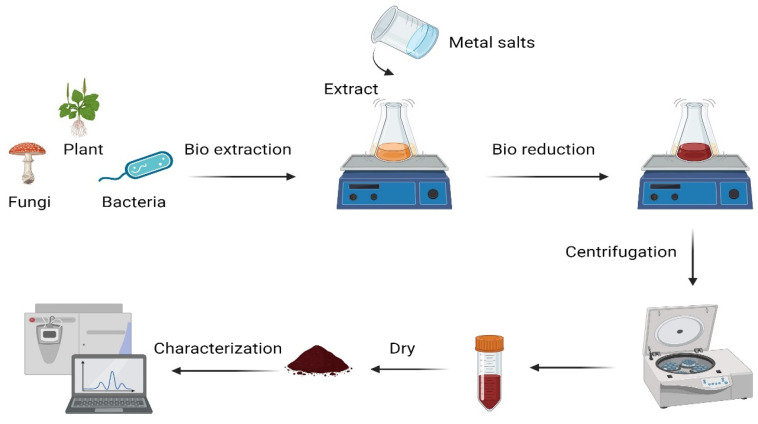
Scheme of green synthesis method for nanoparticles.

The top-down approach is used to transform bulk material into tiny, nanoscale particles. Top-down methods are easy to employ; however, they cannot be used to produce very tiny and regularly shaped particles [112]. The bottom-up approach has become known as the constructive technique. The top-down strategy is completely opposed to bottom-up methods. Atoms and molecules expand and self-assemble to form NPs with different shapes, sizes, and chemical compositions [113,114,115]. Chemical vapor deposition (CVD), biological synthesis, sol-gel, spinning, and pyrolysis are examples of bottom-up techniques [115]. The following three techniques can be utilized to produce NPs, including chemical, physical, and biological [116]. Because it is straightforward, safe, and economical, the biological approach is ideal for creating NPs [115,117], as shown in Table 2. The biological approach is the most efficient and least expensive way to synthesize nanoparticles since it is straightforward and non-toxic [115]. The biological method is straightforward, usually requiring only one step, and good for the environment. In this sense, microbes and various plant parts can be used to create nanomaterials, as illustrated in Figure 1 [118]. The end product is harmless and safe for the environment in nanoparticle form. Using the ideas of green chemistry in nanoscience, safer, more environmentally friendly nanomaterials may be created and processed more quickly. To create and enhance both large- and small-scale processes for creating nanomaterials as well as to use nanomaterials in a variety of industries, green nanotechnology uses green chemistry [119].

The creation of multifunctional nanomaterials that can be used in high-capacity products that may be dangerous to human health and the environment is another goal, as is educating people about the properties of nanoparticles with regard to toxicological concerns [119,120]. It specifically seeks to develop synthesis systems and procedures that can lessen the need for hazardous chemicals while enhancing the efficiency of these already-used synthesis methods. In order to guarantee that the nanoproducts while they are being synthesized are safer, it also provides guidelines for evaluating ecological risks and hazards in connection to design [121]. Numerous species, including fungi, bacteria, and algae, can be used to produce a wide range of nanomaterials from an aqueous solution of metal salts [116]. Living organisms will engage in biomineralization, which includes the production of nanoparticles, by utilizing a protein. For example, magnetotactic bacteria prepare the magnetic particles as a compass to the direction of their chosen habitat at the bottom of the sea in anaerobic conditions using magnetosomes, which are protein-coated for the synthesis of nanosized magnetic iron oxide crystals [122,123]. It has been demonstrated that in vitro synthesis of homogeneous particles with a core diameter of 20–45 nm occurs [124,125]. In spite of this, magnetosomes exhibit potent magnetic properties in therapeutic contexts such as hyperthermia [125,126]. He et al. synthesized 10–20 nm-sized gold nanoparticles extracellularly using microorganisms that produce photosynthesis, such as *Rhodopseudomonas capsulata*. Nicotinamide Adenine Dinucleotide hydride-dependent reductase is a bacterial enzyme that plays a major role in reducing gold ions to gold nanoparticles. They found that the morphology and form of nanoparticles are regulated by the pH of the growth media [127]. Extracellular palladium nanoparticle production from *Pseudomonas* cells from the alpine location was reported by Schlüter et al. [128]. The idea for extracellular Au NPs was proposed by Singaravelu et al. using *Sargassum wightii* algae. Incubation took only 12 h to reach 95% of the yield [129].

**Table 2:** Exploring the anti-HCC properties of nanoparticles derived from natural products.

Nanoparticle	Natural Product	Particle Size (nm)	Shape	IC_50_	The Effect on HCC	Reference
AuNP	*Dendrobium officinale* extract	30 nm	Spherical	200 μg/mL	Anticancer and immune regulation.	[130]
AuNP	*C. militaris* extract	5–25 nm	Face-center-cubic	10–12.5 µg/mL	Activating the gene expression of Bax, Bid, Caspase-3 and Caspase-9.	[131]
AgNP	*Nigella sativa*	10–20 nm	Spherical	7.16 μg/mL	Growth inhibition without harming healthy cells.	[132]
CuONP	*Momordica cochinchinensis* (Lour.) extract	40–80 nm	Cubic	75 μg/mL	Induce apoptosis and ROS.	[133]
AgNP	*Podophyllum hexandrum* Royal leaf extract	14 nm	Spherical	-	Apoptotic effect.	[134]
AgNP	*Amla* extract	188 nm	Spherical and cubic	-	Apoptotic effect.	[134]
AuNP	*Ziziphus spina-christi* leaves	31.26–58.06 nm	Spherical	-	Induce apoptosis in cancer cells.	[135]
AuNP	*Cordia myxa* L. leaves	56.49–89.38 nm	Spherical	-	Induce apoptosis in cancer cells.	[135]
AgNP	*Leucus aspera*	40.67–58.17 nm	Spherical	158 µg/mL	Induce apoptosis in cancer cells.	[136]

## 8. Nanoparticles Used for HCC

### 8.1. Quantum Dots NP

**Figure 2:**
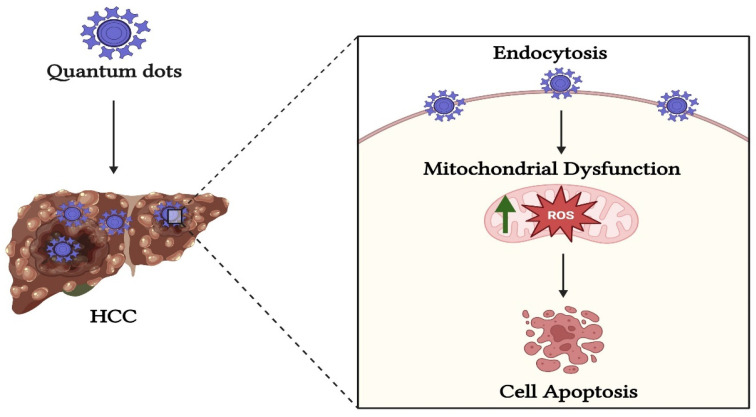
QD induces ROS formation as a promising strategy for treating HCC via apoptosis.

Quantum dots (QDs) are tiny semiconductor particles with small diameters that range from 2 to 10 nm [137]. Furthermore, the distinctive features of QDs are achieved through the precise control of their size, shape, and surface chemistry, which is achieved via the use of modern nanotechnology methods in their synthesis. On the other hand, conventional nanoparticles produced through either bottom-up or top-down approaches may not provide the same degree of control and precision [138,139]. Unique optical features, like size-tunable emission wavelengths and strong photostability, are produced by the quantum confinement phenomenon in QDs [138,139,140]. These properties are not usually seen in larger nanoparticles fabricated via traditional methods [138,140]. They also, display distinctive optical and electrical characteristics as a result of quantum confinement effects. These characteristics make them very beneficial across various fields, including biology, medicine, and quantum technology [137]. QDs have been explored for their potential properties in enhancing drug delivery to HCC [141]. In drug delivery applications, there are significant pharmacological properties improved via QDs; furthermore, their ability to enhance the uptake pathways of cancer drugs [141,142]. Furthermore, QDs can be conjugated to other elements, such as magnetic chitosan, for nano-drug delivery systems that target cancer cells. These QDs can target HCC cells and induce ROS formation in high quantities, as illustrated in Figure 2 [143]. Recent studies reported that carbon quantum dots (CQDs) have shown effective progress in drug delivery systems for various types of cancer therapy, including HCC [143,144]. Cadmium-selenium quantum dots (CdSe QDs) have demonstrated low levels of cytotoxicity, indicating their potential as a promising tool for cancer therapy [145]. Another type of QD is trichrome-tryptophan-sorbitol CQDs, derived from tryptophan [146]. These CQDs showed high green fluorescence in HCC cells, demonstrating their potential for cancer detection and therapy [146].

**Figure 3:**
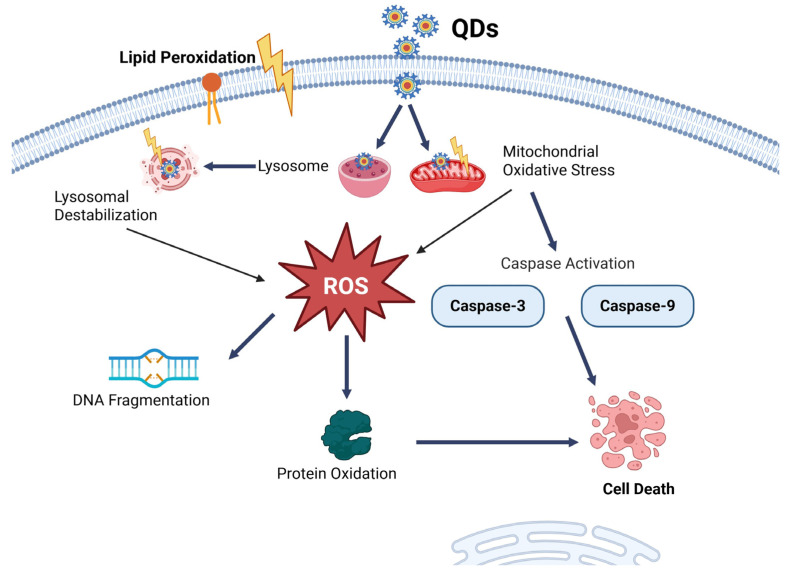
The toxicological pathway of QDs resulting in the formation of ROS leading to cell death.

Although QDs show significant potential for improving HCC therapy via targeted drug delivery and imaging, their application is limited by toxicity concerns mainly associated with their material composition. Furthermore, QDs can produce ROS, which may be damaging to normal cells [147,148,149]. QDs are taken up by cells, leading to lysosomal destabilization and the initiation of oxidative stress via excessive ROS generation [5]. This cascade results in DNA damage, lipid peroxidation, protein oxidation, inflammation, and caspase-mediated apoptosis, which include Caspase-9 and Caspase-3, ultimately leading to cell death, as illustrated in Figure 3 [150,151]. This feature also has applications in therapies such as photodynamic therapy to specifically target cancer cells [147,148,149].

Overall, Progress in surface modification and biofunctionalization is essential for addressing these challenges and ensuring safe and successful therapeutic applications in HCC.

### 8.2. Liposome NP

**Figure 4:**
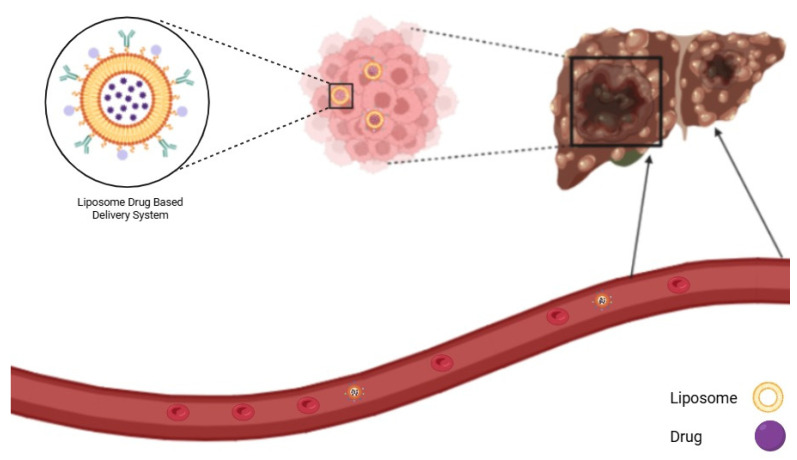
Liposome-based delivery system in HCC.

Liposomes are sealed vesicles with a bilayer structure identical to biological membranes that are made of cholesterol and/or phospholipids as the primary membrane material [152], as illustrated in Figure 4. Because of their excellent biocompatibility, biodegradable non-toxic nature, and non-immunogenicity, liposomes have been used extensively in drug delivery systems [152,153]. Liposomes can simultaneously deliver many therapeutic drugs, including cisplatin and curcumin, to produce a synergistic impact against HCC [154]. This combination amplifies antitumor efficacy and minimizes adverse effects relative to monotherapy [154]. In addition, liposomes can deliver doxorubicin and miR-101 to inhibit tumor development [155]. Also, liposomes can be engineered for targeted delivery, using galactosylated chitosan to specifically target the asialoglycoprotein receptor on HCC, thereby improving the antitumor activity of natural compounds such as oleanolic acid [156]. Liposomes coated with photosensitizers or dyes can be involved in photodynamic and photothermal therapy, enabling a non-invasive treatment approach that successfully targets and ablates HCC cells [157,158]. Liposome nanoparticles present significant promise for HCC treatment due to their capacity to improve drug delivery and specifically target cancer cells. However, challenges such as immunogenicity take place, as liposomal nanoparticles can trigger immune responses and demonstrate off-target toxicity, especially when applied along with immune checkpoint blockade therapies [159]. Additionally, keeping liposomes stable and controlling how they release their encapsulated drug in the acidic environment of tumors presents important challenges that need to be addressed [157,160]. Therefore, liposome nanoparticles loaded with natural products present a promising approach for HCC treatment by improving the administration of drugs, more efficiently targeting tumor cells, and minimizing systemic toxicity. These developments in nanomedicine may result in safer and more efficient treatment alternatives for people with HCC.

### 8.3. Polymeric NP

**Figure 5:**
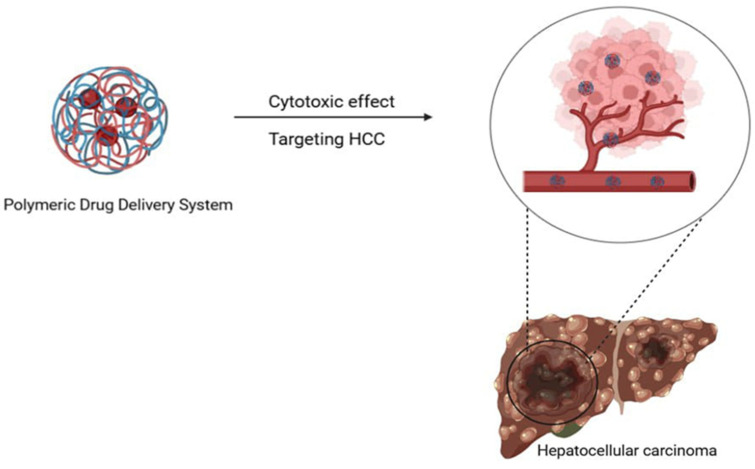
Polymeric delivery system exhibits cytotoxic effects on HCC, highlighting their potential as a targeted therapeutic strategy for liver cancer treatment.

Polymeric nanoparticles (PNPs) are solid, colloidal particles composed of many types of polymers, including biodegradable polymers such as chitosan, collagen, polylactic acid (PLA), and poly(lactic-*co*-glycolic acid) (PLGA) [161]. To increase the bioavailability of drugs that are not highly soluble, PNP can be used to administer the drug orally [162]. This increases the amount of drug at the site of action since the smaller particle size can penetrate capillaries and cells, as illustrated in Figure 5. Furthermore, the use of nanocarriers loaded with natural products has been highlighted as a promising strategy for the treatment of HCC, drawing attention to the fact that natural products exhibit stimulus-responsive drug release and multi-target effects [163]. The use of PNPs that contain natural compounds, such as curcumin, has demonstrated potential in improving the therapy of HCC [164]. By applying these nanoparticles, targeted drug delivery to hepatoma cells is possible due to their enhanced permeability and retention properties [164]. This efficiency results in reduced drug dosage, delayed drug release, and an improved therapeutic effect overall, all while avoiding any apparent adverse effects [21]. However, PNPs often face challenges due to the restricted loading capacity for hydrophobic drugs and inconsistent drug release rates, which may block their therapeutic efficiency [165]. Although PNPs can target-specific areas, they must overcome numerous biological barriers, including systemic, microenvironmental, and cellular barriers, which may differ among patient populations and diseases [166]. Overall, PNPs containing natural products like curcumin have demonstrated the potential to improve HCC therapy by strengthening the targeting, bioavailability, and stability of these drugs. As a starting point for investigating other possible chemotherapeutics, these nanoparticles can give an innovative approach to revolutionary HCC treatment.

### 8.4. Nanozyme

Natural enzymes are well known for their catalytic activity toward specific substrates; however, they require specific circumstances to be stable under harsh conditions like high temperatures [167]. To solve this problem, scientists have identified immobilization techniques using nano- and microscale materials [168]. The combination of these materials in healthcare equipment reflects the characteristics of major biological environments. Numerous materials can enhance these nanomaterials, including artificial polymers, carbon-based nanoparticles, metal-organic frameworks, nanosized protein-based nano-delivery systems, lipid-based nanomaterials, and polysaccharide-based nanoparticles [168]. Hence, nanozymes provide the benefits of natural enzymes in addition to improved stability, ease of storage, and convenient preparation [167]. The early identification of HCC biomarkers through fluorescence imaging poses significant challenges due to the complex physical environment. Human carboxylesterase (CE) is an important biomarker to enable accurate diagnosis of HCC [169]. Current probes for CE demonstrate a sluggish response rate and limited selectivity. The amide group is recognized as CE-responsive points through the application of “the substrate-hydrolysis enzymatic reaction” approach. A series of off–on probes with leaving groups in the amide unit was performed, leading to the identification of the JFast probe, which exhibits the optimum combination of rapid response time along with superior selectivity toward CE. Both in vitro and in vivo experiments demonstrated that the probe activated solely in hepatic cancer cells. This technique offers a precise method for diagnosing HCC [169]. Current investigations indicate that specific metabolic enzymes in cancerous cells assume inappropriate functions, directly affecting gene expression via mechanisms including protein-protein interactions, protein transformations, or protein-kinase behavior [170,171]. For instance, NAD(P)H quinone oxidoreductase-1 interacts with SREBP1, thereby promoting the development and malignancy of HCC, thus contributing to cancer progression [172]. Recognizing the abnormal functions of metabolic enzymes in tumors may provide new strategies for treating neoplasms [173]. One example is magneto-gold nanozymes (AuNC@Fe_3_O_4_) demonstrate photothermal effects and peroxidase-like activity, increasing ROS generation and triggering death in HCC cells via a synergistic mechanism combining photothermal and nano-catalytic therapy [174]. Furthermore, cobalt nanozymes within ferritin nanocages exhibit promise in prognostic diagnosis by differentiating HCC tissues from normal tissues with notable sensitivity and specificity [175]. Also, nanozymes facilitate the detection of HCC-associated microRNA, improving sensitivity by strand displacement amplification and CRISPR-Cas12a systems, enabling visual and colorimetric identification [176]. The staining intensity of these nanozymes is associated with tumor differentiation and patient prognosis. Overall, nanozymes provide various benefits in HCC treatment; challenges remain, such as maintaining biocompatibility and improving delivery systems for clinical use. Further research is required to completely understand their potential in cancer treatment.

## 9. Overcoming Drug Resistance in HCC Using Natural Product-Nanoparticle Combinations

**Figure 6:**
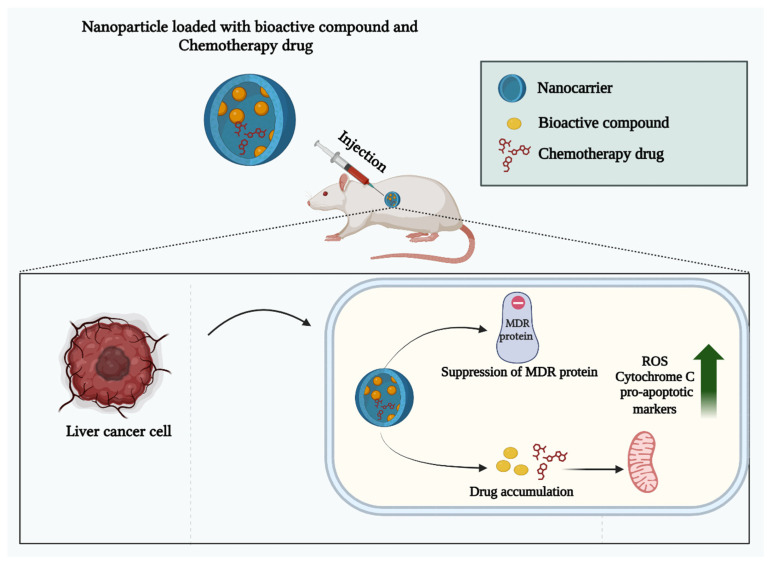
The synergistic effects against MDR in liver cancer cells by up-regulating ROS, Cytochrome C, and pro-apoptotic markers.

The complicated process of MDR in HCC involves several mechanisms, including altered metabolism, decreased drug uptake, aberrant apoptosis, intracellular sequestration, and autophagic signaling, slight changes to the tumor microenvironment [177,178]. Despite remarkable advances in HCC treatment, MDR remains a key problem, limiting the efficacy of chemotherapy and targeted therapy [177,178]. Experimental techniques have been noticed to combat drug resistance, but converting them into favorable clinical results has been challenging [178]. Nanomedicine opens new avenues in reversing MDR [177,179]. Several characteristics make the application of nanomedicine appropriate and potentially effective in combating the resistant phenotype of tumor cells. These include the drug carrier’s small size, the ability to target cancer cells passively or actively, enhanced pharmacokinetics and biodistribution, decreased side effects from the formulation, intrinsic inhibitory activity towards MDR efflux pump proteins, and the feasibility of delivering multiple therapeutic agents in a single formulation [177,180,181]. Various nano-drug delivery strategies, such as dendrimers, liposomes, micelles, polymeric, solid lipid, mesoporous silica, metal nanoparticles, and nanostructured lipid carriers, are highlighted in the current review as ways to circumvent the MDR process [178]. NPs can inhibit key proteins involved in drug resistance, such as Bcl-2, AKT, and P-glycoprotein, thereby enhancing the effectiveness of chemotherapeutic agents [182]. Also, natural compounds have many benefits, including high efficacy, minimal toxicity, and the capacity to address several MDR mechanism routes [183,184]. For MDR reversal agents, natural products increase the tumor cells’ sensitivity to chemotherapeutics and work in concert with them. When natural products and anticancer drugs are delivered together via nanocarriers, the synergistic effects against MDR in tumor cells are maximized [183], as illustrated by Figure 6. Hence, the integration of natural products with nanoparticles presents a synergistic strategy to combat drug resistance [141,179]. Natural products such as celastrol, when formulated with doxorubicin in nanoparticle structure, may increase aqueous solubility, decrease drug dosage, and augment cellular drug accumulation, resulting in apoptosis and autophagy in drug-resistant cells [185]. Despite the significant potential of nanoparticle-based drugs and natural product combinations, challenges persist in optimizing these structures for clinical application.

## 10. Natural Product Delivery Systems for HCC

The utilization of natural products in cancer treatment has garnered significant attention in recent years, primarily due to persistent challenges related to drug resistance particularly in HCC, which is well-recognized for its resistance to conventional therapies [186]. Green drug delivery systems enhance the effectiveness of anticancer treatments by utilizing natural materials, such as plant extracts, marine animals, and microorganisms like fungi and bacteria [186,187]. These materials develop target-specific activity, decrease side effects, and increase in vivo stability and bioavailability [130,187]. Furthermore, natural substances have been used as herbal remedies throughout history for the treatment of HCC, like dietary curcumin and resveratrol loaded with DSPE-PEG_2000_-SP94 nanoparticles and ganoderic acid coated with nano-lipid [188]. In addition, a hepatic cancer cell line (Hep-G2) could be inhibited by spherical AgNPs that were green synthesized by using these plants: *Citrulluss colocynthis*, *Erythrina indica*, *Panax ginseng*, and *Rubus glaucus* Benth [189]. Although various delivery systems exhibit potential, as shown in Table 3, challenges continue to occur, including the need for consistent targeting and the reduction of adverse reactions. Subsequent research should focus on improving these systems for clinical use, maybe using multidisciplinary strategies to improve their effectiveness and safety in HCC treatment.

## 11. Nano-Enabled Personalized Medicine Approaches in HCC

The integration of nanoparticles with natural products presents promising opportunities for personalized medicine in the treatment of HCC [75,199,200]. Theragnostic nanoparticles are a new method that helps in both treatment and diagnosis, allowing for personalized treatment plans by tracking how well drugs are delivered and how tumors respond in real time [201,202]. Furthermore, adaptive therapies can be developed by combining nanoparticles with natural products; these strategies can then be tailored to the specific tumor characteristics and therapeutic response of each patient [203,204]. Nanoparticles can be functionalized with ligands (e.g., antibodies, peptides, aptamers) that specifically bind to HCC receptors such as glypican-3, asialoglycoprotein, or transferrin receptor [163]. For example, sorafenib-loaded nanoparticles that target glypican-3 increase drug accumulation in tumors while preserving normal tissue. An alternative approach is dual-ligand systems. Nanoparticles can be tailored to specific HCC characteristics, including receptor overexpression or mutations in the genome. For instance, dual-targeting nanoparticles are modified to target A54 receptors and nuclear localization signals [205]. Overall, the incorporation of nanoparticles and natural products in HCC treatment provides considerable potential for tailored medicine. This approach improves drug distribution, effectiveness, and safety, providing a customized therapy strategy that targets the complex nature of HCC.

## 12. Limitations in Herbal Medication Based on Nanoparticles

Natural product-based nanotechnology therapy for HCC is limited because of poor aqueous solubility, low cellular absorption, and low bioavailability [32]. Studying the pharmacokinetics of natural products is critical for maximizing therapeutic outcomes and dealing with concerns such as rapid elimination, toxic effects, and inflammation associated with their application [77]. There are major challenges with controlling release in nanoparticles, including non-tunable size, structural heterogeneity, and low release consistency [206]. NPs can trigger or alter the immune response, causing inflammation, immunosuppression, or autoimmune diseases [205,207]. Furthermore, the toxicity of nanoliposomes and QDs in treating HCC is a concern that must be addressed. Both NPs can have adverse effects, including toxicity in normal cells [208,209]. Additionally, nanocarriers can improve the selectivity and delivery of bioactive substances to tumor sites while limiting damage to normal cells. However, challenges related to nanocarrier design and the HCC microenvironment must be eliminated for the effective clinical translation of nanotechnology-based HCC treatment with natural products.

## 13. Conclusions

HCC remains to be considered a primary global health concern, as traditional therapies are frequently constrained by toxicity, drug resistance, and low bioavailability. Herbal medicine, containing various bioactive substances, presents a viable approach for HCC treatment by simultaneously targeting multiple tumor-promoting pathways while minimizing adverse effects. Nanotechnology approaches such as drug delivery systems (DDS) have emerged as promising approaches for the treatment of HCC. Natural products such as Shikonin, Fucoidan, *Nigella sativa*, etc., have shown promising anticancer properties, but their clinical application is often limited by low bioavailability, poor solubility, and potential adverse effects. Encapsulating or attaching natural products to nanoparticles can improve their pharmacokinetic profile, allowing for controlled release, targeted delivery, overcoming drug resistance, and enhanced therapeutic efficacy. The conjugation of nanoparticles, such as quantum dots and nanoliposomes, with natural products offers a potential strategy to improve effectiveness and reduce the adverse effects of these natural products. Despite these advances, problems such as scalability, long-term safety, and clinical translation remain. Future research should optimize DDS for herbal substances, perform thorough preclinical and clinical trials, and develop personalized medicine strategies.

## Figures and Tables

**Table 3:** Delivery systems of natural products for HCC treatment.

Nanoparticle	Natural Product	Size of Nanocomposite	Characterization Techniques	Model	Advantages	Reference
Liposome of biocompatible matrix DSPE-PEG_2000_ decorated with short peptide ligand SP94	Curcumin and resveratrol	<200 nm	TEM, DLS, and UV–VIS	In vivo and in vitro	- Increasing tumor apoptosis by increasing ROS generation. - Targeting peptide SP94 improves drug accumulation in tumors via the EPR effect.	[21]
*Morus nigra* L.-derived lipid nanoparticles (MLNPs)	Fresh leaves of *Morus nigra* L.	Approximately 100 nm Hydrodynamic size 162.1 nm	AFM, DLS, and TEM	In vivo and In vitro	- MLNPs caused cell cycle arrest at the G0/G1 phase and induced apoptosis. - They also triggered a surge in intracellular ROS levels and significantly inhibited the proliferation and migration of hepatoma cells.	[190]
Galactose-modified PEGylated liposomes (C-GPL)	Celastrol	139.4 ± 2.7 nm	TEM, and DLS	In vivo and In vitro	- Enhancing its cellular uptake and reducing its side effects. - Effectively inhibited the development of HCC by suppressing AKT activation. - Inducing cell apoptosis and retarding cell proliferation	[191]
Naringenin nanoparticles (NARNPs)	Naringenin	54.96 ± 18.6 nm TEM 31.79 ± 6.8 nm SEM	SEM, TEM, FT-IR, and XRD	In vitro	- NARNPs effectively inhibit cell proliferation and induce apoptosis. - No cytotoxic effects on normal cells.	[192]
SeNPs and AgNPs	Berberine	171.5 ± 4.2 nm	DLS	In vitro	- Inhibit the growth and migration of HepG2 by triggering oxidative stress and apoptotic cascades.	[193]
Glucan nanoparticles	Curcumin	111.0 ± 49.0 nm	DLS, ELS, and Cryo-SEM	In vitro	- Induce ROS in HepG2 cells, impacting oxidative stress. - NPs modulate cancer therapy by affecting inflammatory chemokines like RANTES. - Encapsulation enhances *curcumin*’s bioactivity, affecting cellular responses.	[194]
Fe_2_O_3_/Starch/Polyvinyl alcohol nanocarrier (Fe_2_O_3_/S/PVA NC)	Quercetin (QC)	240–340 nm	FTIR, XRD, FE-SEM, DLS, and VSM	In vitro	- Suppressing HepG2 cancer cells by targeted delivery of QC drug. - Fe_2_O_3_/S/PVA caused 6 % of HepG2 cells to early apoptotic death.	[195]
AuNPs	Polystyrene polysaccharide extracted from *Polygonatum*	91–459 nm	UV–VIS, TEM, AFM, FTIR, DLS, and EDX	In vitro and In vivo	- Enhancing immunoregulation. - Higher cancer suppression rates.	[196]
AuNPs	*Panicum maximum* leaf extract	45∼50 nm	TEM, and UV–Vis	In vitro	- Induce cell apoptosis and ROS.	[197]
CuONPs	*Neem* leaves extract *Azadirachta indica*	15∼16 nm	XRD, FTIR, and UV–VIS	In vitro	- Decline of cell viability caused by DNA destruction.	[198]

## Data Availability

Not applicable.

## Abbreviations

HCC	Hepatocellular carcinoma
PKM2	Pyruvate kinase type M2
Hep-G2	Hepatic cancer cell line
PYCR1	Pyroline-5-carboxylate reductase 1
ROS	Reactive oxygen species
GTCs	Green Tea Catechins
EGCG	Epigallocatechin-3-gallate
AFP	Alpha-fetoprotien
MRI	Magnetic resonance imaging
CT	Computed tomography
MDR	Multidrug resistance
ICG	Indocyanine Green
NPs	Nanoparticles
TKIs	Tyrosine kinase inhibitors
SFN	Sorafenib
QDs	Quantum dots
TQ	Thymoquinone
VEGF	Vascular endothelial growth factor
CdSe QDs	Cadmium-selenium quantum dots
PNPs	Polymeric nanoparticles
PLA	Polylactic acid
CVD	Chemical vapor deposition
PVA	Polyvinyl alcohol
NARNPs	Naringenin nanoparticles
QC	Quercetin
C-GPL	Galactose-modified PEGylated liposomes
MLNPs	*Morus nigra* L.-derived lipid nanoparticles
PLGA	Poly(lactic-*co*-glycolic acid)
